# Deciphering and modulating G protein signalling in *C. elegans* using the DREADD technology

**DOI:** 10.1038/srep28901

**Published:** 2016-07-27

**Authors:** Simone Prömel, Franziska Fiedler, Claudia Binder, Jana Winkler, Torsten Schöneberg, Doreen Thor

**Affiliations:** 1 Institute of Biochemistry, Molecular Biochemistry, Medical Faculty, University of Leipzig, 04103 Leipzig, Germany

## Abstract

G-protein signalling is an evolutionary conserved concept highlighting its fundamental impact on developmental and functional processes. Studies on the effects of G protein signals on tissues as well as an entire organism are often conducted in *Caenorhabditis elegans.* To understand and control dynamics and kinetics of the processes involved, pharmacological modulation of specific G protein pathways would be advantageous, but is difficult due to a lack in accessibility and regulation. To provide this option, we designed G protein-coupled receptor-based designer receptors (DREADDs) for *C. elegans*. Initially described in mammalian systems, these modified muscarinic acetylcholine receptors are activated by the inert drug clozapine-N-oxide, but not by their endogenous agonists. We report a novel *C. elegans*-specific DREADD, functionally expressed and specifically activating G_q_-protein signalling *in vitro* and *in vivo* which we used for modulating mating behaviour. Therefore, this novel designer receptor demonstrates the possibility to pharmacologically control physiological functions in *C. elegans*.

Designer receptors exclusively activated by designer drugs (DREADDs) have been developed as a tool to study and to specifically manipulate G-protein signalling *in vivo*^1^,^2^. This technology for the selective control of signalling cascades has been found to be invaluable not only to investigate the impact of specific G-protein signalling on distinct physiological processes but also to control cellular functions in a cell-type specific manner. Besides DREADDs as pharmacogenetic tools also optogenetic methods have been generated. Both approaches are non-invasive and based on modified receptors which can be specifically activated – DREADDs exclusively by synthetic ligands and optogenetic tools only by light.

The first generation of DREADDs was based on muscarinic acetylcholine receptors which have been altered by two point mutations within transmembrane domains 3 and 5 to inhibit activation by the endogenous agonist acetylcholine (ACh) but allowing stimulation by the inert compound clozapine-N-oxide (CNO)^1^. Further modification allowed for specific activation of G_q_, G_s_, or G_i_ signalling pathways, respectively^2^. These DREADDs are called rM3Dq, rM3Ds, and hM4Di by convention^3^. They have been extensively pharmacologically characterized^4^ and widely used to specifically activate and inactivate for instance neuron populations *in vivo* by employing the G_q_- and the G_i_-specific DREADDs, respectively^5^. DREADDs have also been involved to study neuronal impact on fear memory, Parkinson disease, Down syndrome, and the role of glial cells *in vivo*^6^,^7^,^8^,^9^. In the periphery, the DREADD technology has been applied to study metabolic functions in islet β-cells and hepatocytes^2^,^10^.

Despite the power and simplicity in the use of DREADDs many physiological processes and functions associated with specific signalling cascades are not easy to delineate in mammals due to the complexity of the organisms. The application in less complex systems would be highly advantageous to overcome these limitations. Recently, the DREADD technology was transferred to *Drosophila melangoster* showing that mammalian DREADDs can be efficiently expressed in the fruitfly and modulate physiological functions linked to G-protein pathways^11^. The use of mammalian DREADDs in other organisms also raises the interesting question whether organism-specific DREADDs can be generated likewise. The nematode *Caenorhabditis elegans*, a very simple model organism frequently employed for the investigation of various biological questions, is highly suitable for the dissection of the impact of signalling cascades. So far only optogenetics have been successfully utilised as a tool for the manipulation of some signalling cascades in the nematode^12^,^13^,^14^. However, G protein-signalling pathways are not well addressed by non-genetic tools.

Here, we show that the DREADD technology can be applied in *C. elegans* using a newly designed DREADD based on the nematode muscarinic receptor GAR-3b. In-depth pharmacological characterisation *in vitro* revealed that the DREADD specifically activates G_q_ signalling. In the nematode the DREADD is able to modulate physiological functions upon stimulation with CNO *in vivo*.

## Results

### Mammalian DREADDs are not active in *C. elegans*

One prerequisite for the use of DREADDs in *C. elegans* is that the synthetic ligand CNO utilised to activate these receptors does not have any adverse effects on nematodes. To elucidate the influence the compound has on *C. elegans* fertility, development, viability, and the neuronal system as well as certain aspects of behaviour we treated wild-type nematodes in liquid culture with varying concentrations of CNO and assayed brood size, individuals reaching adulthood, lifespan, locomotion, pharyngeal pumping, egg laying and sensitivity to aldicarb. However, none of the parameters was affected (Supplementary Fig. 1) indicating that the compound does not have any major side effects on *C. elegans*.

**Figure 1:**
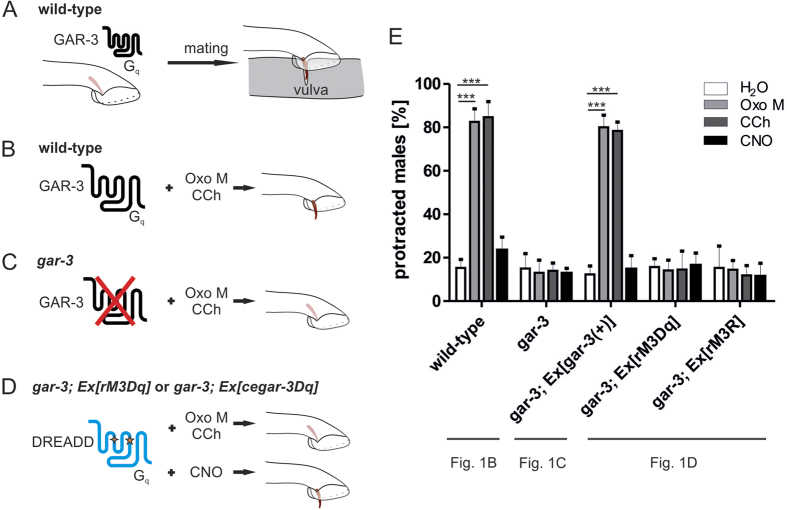
The mammalian rM3Dq DREADD and rM3R are not functional in spicule protraction, as a model for DREADD activation in *C. elegans*. (**A–D**) Schematic representation of spicule protraction and theoretical model of GAR-3 and DREADD activation in this context, that all DREADD characterisations are based on (see Figs 1E and 4F). (**A**) GAR-3 mediates a G_q_-protein signal which is involved in spicule protraction of the male nematode during mating culminating in insertion of the spicule into the hermaphrodite’s vulva. (**B**) GAR-3 can be activated by Oxo M and CCh to induce spicule protraction involving the G_q_ protein-signalling cascade. This effect is independent of the presence of a hermaphrodite. (**C**) In the absence of GAR-3 neither Oxo M nor CCh trigger spicule protraction. (**D**) DREADDs activating the G_q_ protein-signalling cascade specifically trigger spicule protraction upon stimulation with CNO, but not with Oxo M or CCh. Such DREADDs can be rM3Dq or a *C. elegans*-specific DREADD (cegar-3Dq) in *gar-3*-deficient males. (**E**) Protraction rate in male nematodes containing mammalian DREADD constructs. Males were incubated with 100 mM Oxo M, 10 mM CCh, 2 mM CNO or H_2_O as negative control. Subsequently, spicule protraction was scored and protraction rates calculated in respect to the total male count. Wild-type worms (*pha-1*(*e2123*)*; him-5* (*e1490*))*, gar-3* (*pha-1*(*e2123*)*; him-5* (*e1490*) *gar-3*(*gk305*)) and transgenic *gar-3; Ex*[*gar-3*(+)] males served as controls. Data are shown as mean ± SD. ***p < 0.001; n ≥ 250. Indicated below each set of columns is the schematic model related to the respective data.

As mammalian DREADDs have been shown to be a useful tool for analyses in *Drosophila*, suggesting that they are able to activate distinct G-protein cascades in vertebrates and invertebrates likewise^11^, we tested these receptors in *C. elegans*. To investigate the functionality of mammalian DREADDs in the nematode, systems are required in which the physiological implications of G_s_, G_q_, and G_i_ protein-mediated pathways, respectively, are well understood. Protraction of copulatory spicules from the tail of the male nematode is dependent on a G_q_ signalling cascade^15^,^16^ and thus, offers one suitable read-out. Spicule protraction occurs during mating when the spicule is inserted into the hermaphrodite’s vulva (Fig. 1A)^15^,^17^. The *C. elegans* homolog of the muscarinic acetylcholine receptor M_3_, GAR-3, is a G protein-coupled receptor (GPCR) involved in controlling this process. GAR-3 has been shown to activate a G_q_ cascade similar to its mammalian homolog^18^,^19^,^20^ and even triggers G-protein signalling in mammalian cells^20^ indicating that this receptor/G_q_-protein cascade is evolutionary well preserved. Thus, we speculated that the DREADD (rM3Dq) which is based on the rat M_3_ receptor (rM3R) can activate G_q_ signalling in the nematode. The *C. elegans* strain null for *gar-3*, *gar-3*(*gk305*) displayed a spicule protraction defect (Fig. 1C,E Supplementary Tab. 1A) which was in concordance with previous studies^16^. It has been shown that carbachol (CCh) and oxotremorine M (Oxo M) activate GAR-3^16^,^21^ and thus, induced spicule protraction in wild-type males (Oxo M 82.5 ± 3.3%; CCh 84.7 ± 7.8%), independently of a hermaphrodite being present (Fig. 1B,E). Consistently, we did not observe this effect in *gar-3*(*gk305*) mutants (Oxo M 10.6 ± 4.7%; CCh 12.8 ± 2.4%) (Fig. 1C,E, Supplementary Tab. 1A). We tested whether G_q_ signalling induced by rM3Dq signalling rescues this defect by expressing the DREADD rM3Dq under the control of the *gar-3* promoter in the *gar-3* null background and stimulating the nematodes with CNO. However, we were unable to obtain any rescue (15.1 ± 5.1%) (Fig. 1E, Supplementary Tab. 1A). The same effect was seen in transgenic lines using the unmodified rM3R driven by the *gar-3* promoter (Oxo M 14.0 ± 4.0%; CCh 13.2 ± 5.4%) (Fig. 1E, Supplementary Tab. 1A). Due to this lack of functionality we investigated expression of the *yfp*-tagged receptors by using fluorescent imaging techniques. However, we were unable to detect any protein (Supplementary Fig. 2) despite obtaining several transgenic lines containing receptor DNA (Supplementary Tab. 2). Likewise, no expression was observed for the other two mammalian DREADDs for G_s_-protein signalling (rM3Ds) and for G_i_-protein signalling (hM4Di) (data not shown).

Therefore, we conclude that the lack of activity of mammalian DREADDs in *C. elegans* is likely due to difficulties in properly expressing or processing the receptors.

### Generation of a *C. elegans*-specific DREADD

**Figure 2:**
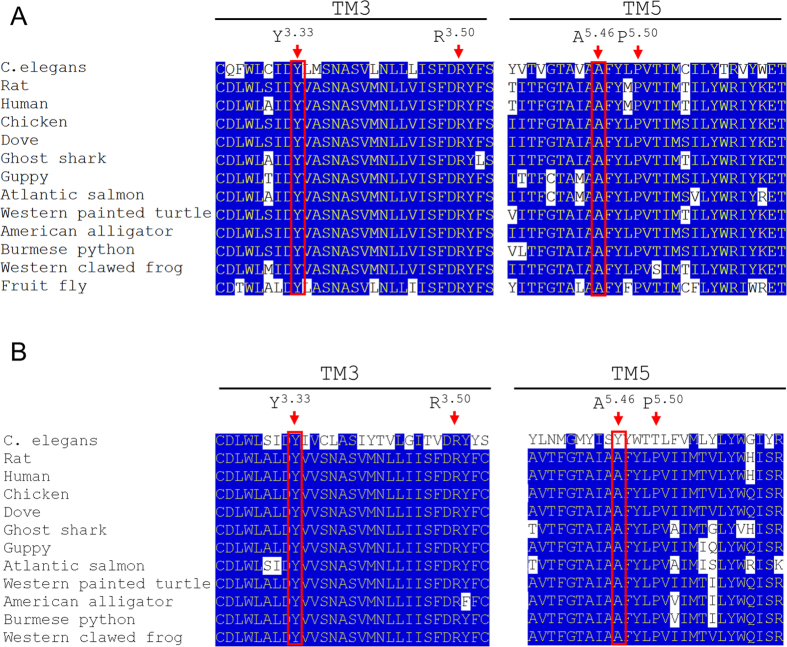
Amino acid alignment of transmembrane domains 3 and 5 of muscarinic acetylcholine receptor orthologs. (**A**) Sequences of mAChR type 3 from 13 different species were retrieved from publically available databases such as NCBI/GenBank. Alignment was performed using the Clustal W alignment^48^. Shown is the amino acid sequence of transmembrane domain 3 and 5. Conserved residues are shaded in blue. Highlighted in red are the highly conserved tyrosine Y3.33 (Y146) and alanine A5.46 (A237) which are mutated in mammalian DREADDs. (**B**) Analogously, alignment of mAChR type 2 was performed. While Y3.33 is also conserved in *C. elegans* GAR-2, amino acid 5.46 differs from other muscarinic orthologs.

Since mammalian DREADDs did not seem to be properly expressed in *C. elegans*, we set out to generate a nematode-specific DREADD for modulation of G protein-signalling pathways in the worm. We sought to base this modified receptor on a muscarinic acetylcholine receptor similar to the mammalian DREADDs. Three G protein-linked acetylcholine receptor genes are known in *C. elegans* (*gar-1, gar-2, gar-3*) with GAR-2 being described to bind G_i_/G_o_ proteins and GAR-3 being a G_q_-coupling receptor^21^,^22^. However, GAR-1 and GAR-2 differ in their pharmacological properties from mammalian muscarinic acetylcholine receptors^21^,^23^,^24^, whereas GAR-3 is pharmacologically very similar to mammalian M3R and activates the G_q_ signalling cascade upon stimulation with ACh or CCh^19^,^20^,^21^. Further, alignment analyses revealed an overall amino acid sequence identity of GAR-3 to rM3R of 32.8%. Two splice variants, GAR-3a and GAR-3b, have been described with GAR-3b being the predominantly occurring form. The two isoforms only differ in 26 amino acid length within the 3^rd^ intracellular loop (ICL)^25^. Also, amino acids Y3.33 and A5.46 (universal amino acid numbering system^26^), which are widely conserved among muscarinic acetylcholine receptors, are also present in the *C. elegans* GAR-3b (Fig. 2A), but only Y3.33 is conserved in *C. elegans* GAR-2 (Fig. 2B). Therefore, we chose GAR-3b for generation of a *C. elegans*-specific DREADD and – analogously to mammalian DREADDs – mutated amino acids Y3.33 (Y146C) and A5.46 (A237G). The DREADD was N-terminally tagged with an HA-epitope to allow for detection using commercially available antibodies.

### Pharmacological characterisation of ceGAR-3Dq

**Figure 3:**
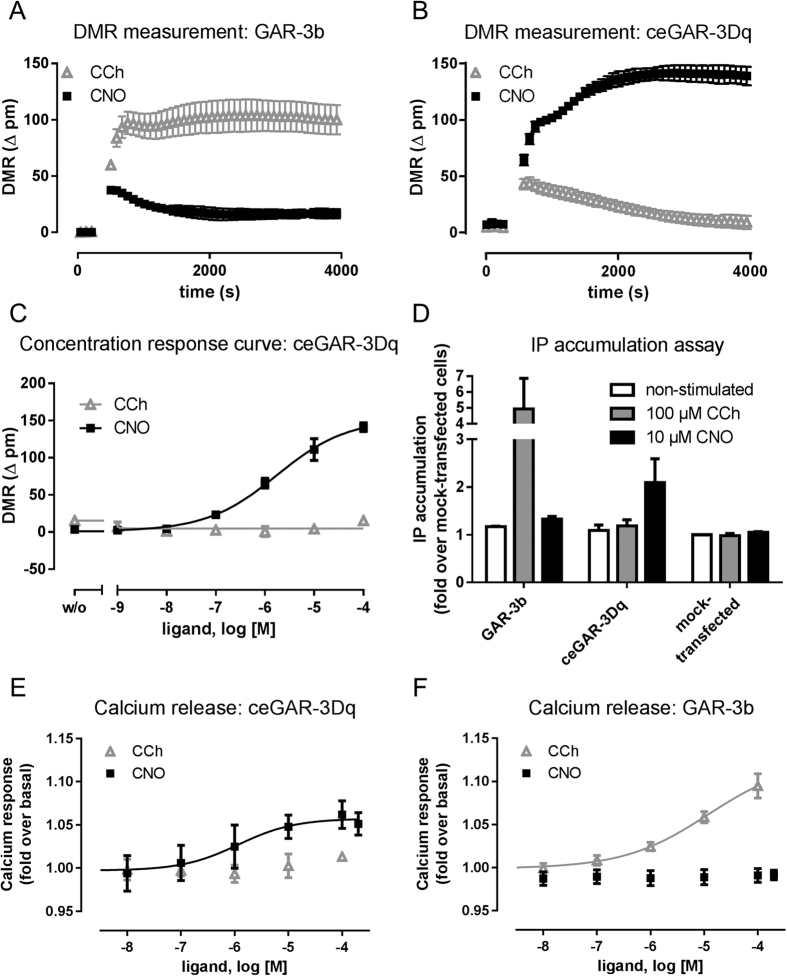
Pharmacological characterisation of ceGAR-3Dq. Agonist specificity was determined using dynamic mass redistribution. (**A**) GAR-3b is stimulated by 100 μM CCh, whereas 10 μM CNO does not stimulate the receptor. (**B**) 100 μM CCh cannot stimulate ceCAR-3b, but the DREADD is stimulated by 10 μM CNO. (**C**) The DREADD agonist CNO activates ceGAR-3Dq in a concentration dependent manner whereas the muscarinic agonist CCh does not have an effect on receptor activity. Given are one of three representative experiments performed in triplicates. (**D**) Second messenger assays reveal G_q_-protein coupling of GAR-3b and ceGAR-3Dq. Transfected cells were incubated with media (non-stimulated), 100 μM CCh, or 10 μM CNO. CCh-stimulation of GAR-3b leads to a robust increase in IP formation, but ceGAR-3Dq is only activated by CNO. Given is the mean ± SD of three to four independent experiments performed in triplicates. (**E**) Calcium release was measured in ceGAR-3Dq transfected cells after stimulation with CCh and CNO. While CCh does not trigger Calcium release, CNO results into a concentration-dependent Calcium release. (**F**) GAR-3b transfected cells release Calcium after stimulation with CCh but not CNO. Given is the mean ± SEM of three independent experiments performed in duplicates.

The modified GAR-3b was pharmacologically characterized in respect to agonist and coupling specificity. Due to the high conservation of the three major G proteins G_q_, G_i_, and G_s_ among metazoan species analyses of functional G protein-coupling of receptors from *C. elegans* or other invertebrate species can be performed using mammalian systems^27^,^28^,^29^ with the results being transferable to the original system. Thus, we used transiently transfected COS-7 cells, as DREADD cell surface expression was highest compared to CHO-K1 and HEK-293GT cells (Supplementary Fig. 3A). Utilising label-free dynamic mass redistribution (DMR) experiments, we demonstrated that GAR-3b is only activated by CCh (Fig. 3A) whereas the receptor mutant (ceGAR-3Dq) is indeed a DREADD solely activated by the inert drug CNO but not by the muscarinic agonist CCh (Fig. 3B). CNO activated the DREADD in a concentration-dependent manner with an EC_50_ value of 3.5 μM, whereas the muscarinic agonist CCh did not have an effect on receptor activity (Fig. 3C). To analyse the coupling specificity, further experiments involved detection of second messengers such as inositol phosphate (IP) as a read out for receptor activation. Consistent with the DMR measurements IP accumulation assays showed an increase in IP formation upon CCh stimulation of GAR-3b but not upon CNO treatment (Fig. 3D). In contrast, DREADD-mediated IP formation occurs only after stimulation with CNO. Further, measurement of calcium release also demonstrates the concentration-dependent activation of the DREADD-receptor upon CNO treatment (Fig. 3E), whereas GAR-3b is only activated by CCh (Fig. 3F). Neither of these compounds has any effect on mock-transfected cells demonstrating the receptor-specificity (Supplementary Fig. 3B).

This is consistent with previous studies where GAR-3-transfected CHO-K1 cells were shown to accumulate inositol phosphates and release Ca^2+^ upon CCh treatment^19^,^21^,^25^,^30^. Further, these elevated Ca^2+^ levels have been shown to stimulate cAMP production independent of G_s_ protein-signalling^20^. Also, it was demonstrated that GAR-3 is linked to G_i_–protein coupling in cell culture experiments^20^,^25^. Thus, we tested for potential G_s_- or G_i_-protein coupling. Firstly, we applied second messenger assays to measure cAMP accumulation with and without pre-incubation of the adenylyl cyclase stimulator forskolin. Neither stimulation of GAR-3b nor of ceGAR-3Dq resulted in a change of cAMP accumulation (Supplementary Fig. 4A,B). However, it has to be noted that expression levels of GAR-3b and ceGAR-3Dq were rather low. Therefore, the lack in identifying Ca^2+^-mediated cAMP formation might be due to a lack of sensitivity. In fact, in a more sensitive CRE-based reporter gene assay an increase of cAMP was observed (Supplementary Fig. 4C,D). The EC_50_ values are similar to those obtained in DMR measurements (CNO at ceCAR-3Dq: 1.6 μM; CCh at GAR-3b: 4.9 μM) (Supplementary Tab. 3). For further validation of the G protein-coupling properties of GAR-3b, DMR measurements were performed with pertussis and cholera toxin to test for coupling to other G protein-signalling pathways. Neither cholera toxin nor pertussin toxin, a strong inhibitor of G_i_-protein signalling^31^, altered signalling on CCh-stimulated GAR-3b transfected cells suggesting no involvement of toxin-sensitive G_s_- or G_i_-protein pathways (Supplementary Fig. 4F,G). These results indicate exclusive G_q_–protein coupling of the DREADD and according to the suggested nomenclature the DREADD was therefore named ceGAR-3Dq^3^.

### G_q_-protein signalling triggered by ceGAR-3Dq modulates spicule protraction behaviour in male nematodes

**Figure 4:**
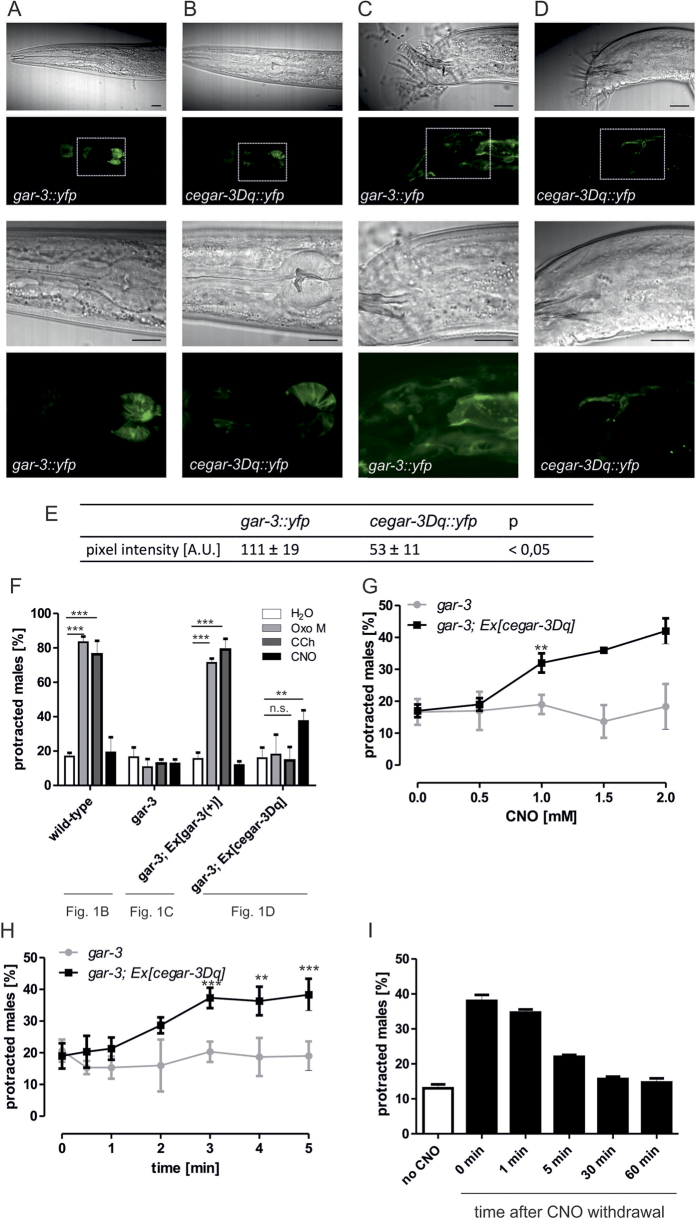
Cegar-3Dq is functional upon stimulation with CNO in *C. elegans*. (**A–D**) Expression and protein localisation of *cegar-3Dq::yfp* is indistinguishable from *gar-3::yfp*, both driven by the *gar-3* promoter. Expression was detected in the pharynx (A, B; 1./3. row: DIC, 2./4. row: fluorescent image) and in the male tail (C, D; 1./3. row: DIC, 2./4. row: fluorescent image). Localisation of the DREADD appears to be similar to the one of GAR-3. Scale bars = 10 μm. (**E**) Analyses of expression levels by quantification of intensities reveals that *cegar-3Dq::yfp* expression is significantly weaker than *gar-3* expression. For quantification, images of the pharynx from different *cegar-3Dq::yfp* expressing strains were taken and analysed in comparison with images from nematodes carrying *gar-3::yfp*. (**F**) Protraction rate in male nematodes containing the *cegar-3Dq* construct. Males were stimulated with 100 mM Oxo M, 10 mM CCh, 2 mM CNO or H_2_O as negative control. Subsequently, spicule protraction was scored. Wild-type worms (*pha-1*(*e2123*)*; him-5* (*e1490*))*, gar-3* (*pha-1*(*e2123*)*; him-5* (*e1490*) *gar-3*(*gk305*)) and transgenic *gar-3; Ex*[*gar-3*(+)] males served as controls. Data are shown as mean ± SD. n.s. not significant; **p < 0.01; ***p < 0.001; n ≥ 300. (**G**) Spicule protraction rates are dependent on the concentration of CNO. Male nematodes were incubated with the indicated concentrations of CNO and spicule protraction was scored subsequently. Nematodes deficient for *gar-3* (*pha-1*(*e2123*)*; him-5* (*e1490*) *gar-3*(*gk305*)) served as negative control. Data are shown as mean ± SD, **p < 0.01; ***p < 0.001; n ≥ 200. Indicated below each set of columns is the schematic model related to the respective data. (**H**) Treatment duration does not have an effect on spicule protraction rate after 3 minutes. Male nematodes were incubated with 2 mM CNO and spicule protraction was scored. As negative control, nematodes deficient for *gar-3* (*pha-1*(*e2123*)*; him-5* (*e1490*) *gar-3*(*gk305*)) were used. Data are shown as mean ± SD, **p < 0.01; ***p < 0.001; n ≥ 200. (**I**) The effect of DREADD activation is reversible. Transgenic *gar-3; Ex*[*gar-3*(+)] males were stimulated with 2 mM CNO. After 3 minutes the spicule protraction rate was determined (time point termed “0 minutes after CNO withdrawal”) and nematodes were withdrawn from the drug by transferring them into M9. Subsequently, spicule protraction was measured at distinct time points. Data are shown as mean ± SD, n ≥ 150.

We next tested the applicability of the GAR-3-based DREADD ceGAR-3Dq *in vivo*. To ensure that the receptor is functional we first assessed expression of *ceGAR-3Dq::yfp* based on genomic *gar-3* driven by the *gar-3* promoter which is indistinguishable from *gar-3::yfp*, albeit appearing to be generally weaker (Fig. 4A–E). Subsequently, the capability of the DREADD to activate a G_q_ protein-signalling cascade when stimulated with CNO was investigated by quantifying spicule protraction movements (see Fig. 1D). Transgenic *gar-3*(*gk305*)*;Ex*[*cegar-3Dq*] nematodes displayed no spicule protraction upon treatment with Oxo M or CCh (Oxo M 17.9 ± 11.5%; CCh 13.6 ± 4.8%) similar to the effect seen in untreated males (16.8 ± 2.1%) (Figs 1B–D and 4F, Supplementary Tab. 1B), indicating that the DREADD is not activated by GAR-3 muscarinic agonists. However, spicule protraction was triggered upon stimulation with the synthetic DREADD agonist CNO (37.4 ± 6.3%) (Figs 1D and 4F, Supplementary Tab. 1B). These data are consistent with the pharmacological analyses *in vitro* showing that ceGAR-3Dq is a DREADD. To optimise the observed effect of ceGAR-3Dq-activated G_q_-protein signalling on spicule protraction, a dose-response curve using increasing concentrations of CNO was conducted (Fig. 4G). However, due to the limited solubility of the compound, a maximal concentration of 2 mM CNO was tested. Unfortunately, stimulation did not reach saturation and thus, we were unable to determine an EC_50_ value. Approximately 40% of males protracted their spicules at this concentration. Activation of the DREADD and thus, spicule protraction was already detected 2 minutes after start of CNO treatment. Treatment duration did not have any influence on the extent of spicule protraction (Fig. 4H).

We next asked whether DREADD activation can be temporally controlled. Upon removal of *gar-3*(*gk305*)*;Ex*[*cegar-3Dq*] nematodes from CNO the spicule protraction rate decreased over time (Fig. 4I). Approximately 30 minutes post drug withdrawal spicule protraction reached the same level as observed in untreated controls, suggesting that DREADD activation is reversible. These results indicate that ceGAR-3Dq can be specifically activated by CNO *in vivo* and is able to mediate a G_q_-protein signalling cascade. This activation can be temporally controlled.

## Discussion

GPCRs are the largest family of cell surface receptors and the signals they mediate are involved in nearly all physiological functions^32^. Thus, gaining an in-depth understanding of their signalling modes and the ability to specifically modulate the signalling pathways has been intensively studied. However, determining the impact of a single type of receptor and its signalling pathways in a distinct cell type or tissue is virtually impossible as a single receptor is usually present in more than one cell type and endogenous agonists activate more than one receptor^33^. For the same reasons it is problematic to modulate distinct signalling cascades in a specific cell type in order to alter or control cellular functions. To overcome those limitations pharmacogenetic techniques in the form of designer receptors have been developed^1^. These so-called DREADDs are only activated by the inert compound CNO but not by the endogenous ligand ACh and thus, are a valuable tool to specifically activate G protein-signalling pathways *in vivo*. Combining this technique with the use of simple model organisms would render a powerful tool for the investigation and control of cell-type specific signalling.

In the present study, we generated a DREADD for the nematode *C. elegans* based on the G protein-linked acetylcholine receptor *gar-3*. This DREADD couples to the G_q_ protein-signalling cascade similar to GAR-3^21^ and is activated exclusively by CNO. As a proof of principle, we provide functional data that the DREADD can be employed for spatiotemporal control of signalling in the nematode.

The DREADD technology is highly suitable for the use in the model organism *C. elegans* and genetically amenable so that DREADD constructs can be easily introduced. Further, we have shown that the synthetic compound CNO which activates DREADDs can be administered by feeding or soaking nematodes without having any major detrimental effects.

Although expression of selected mammalian GPCRs in *C. elegans* has been shown to be generally possible for some receptors^34^, introduction of mammalian DREADDs rM3Dq, rM3Ds, and hM4Di as well as rM3R in the nematode did not render any functional receptors, despite their high sequence and functional conservation among various species. Our data indicate that these receptors are possibly not properly expressed in *C. elegans*. Several factors may contribute to this fact. As transgenic lines containing the receptors were obtained, a potential toxic effect of the constructs or their gene products can be ruled out. However, processing or membrane targeting of the mammalian proteins might be an issue. Alternatively, it is conceivable that certain co-factors or interaction partners required for functional expression are not present in *C. elegans*. Even though the codons of the mammalian cDNA sequences were reviewed prior to construct generation and no overly problematic ones were identified, it cannot be excluded that non-optimal codon usage might account for this effect. It is, however, conceivable that these receptors are transcribed but not processed properly in nematode cells.

As the technology of mammalian DREADDs was not easily transferable to *C. elegans*, we sought to generate a nematode-specific one and have chosen the M3R homolog GAR-3. While the ceGAR-3Dq transgene used for the *in vivo* studies is based on the genomic locus of *gar-3*, cDNA of the predominantly occurring GAR-3b isoform was used for its initial pharmacological characterisation. This isoform does not differ from GAR-3a in its pharmacological properties, which is also similar to rat M3R where large parts of the 3^rd^ intracellular loop can be deleted without changing receptor functionality^35^. In both cases we inserted two point mutations according to mammalian DREADDs in transmembrane domains 3 and 5 of the receptor (Y146C/A237G in GAR-3) for generation of the novel DREADD. DMR measurements demonstrated that the modified GAR-3b is indeed a DREADD, only activated by CNO but not by the muscarinic agonist CCh (Fig. 3). The EC_50_ value for CNO activation determined in DMR measurements is 3.5 μM (pEC50: 5.46 ± 0.43) which is about 100-fold higher than for the mammalian DREADD rM3Dq determined in yeast or cell culture experiments^1^,^2^,^4^,^36^.

This lower potency of CNO at the *C. elegans* DREADD is probably due to the lower potency of agonists at GAR-3b compared to rM3R (Supplementary Tab. 3). *In vitro* analyses of coupling properties of GAR-3b and ceGAR-3Dq revealed a robust G_q_-mediated signalling with a Ca^2+^-dependent increase in cAMP^37^. Similar results have been obtained in previous studies^19^.

Although it cannot be easily proven that the GAR-3-based DREADD exclusively activates G_q_ signalling cascades especially as there are 21 Gα subunits in *C. elegans* with 17 being uncharacterized in regards to their signalling abilities as no clear homologs exist^38^,^39^, our functional assays point towards ceGAR-3Dq activating predominantly G_q_ signalling.

*In vivo* analyses revealed that the GAR-3b-derived DREADD ceGAR-3Dq is able to mediate a G_q_-protein signalling cascade upon stimulation specifically with CNO but not with CCh or Oxo M. Activation of ceGAR-3Dq led to spicule protraction in males lacking *gar-3* showing that the DREADD can complement the GAR-3-signalling cascade when activated. The detected effect is approximately 40% while 80% are achieved when re-introducing the *gar-3* genomic sequence into *gar-3*-deficient nematodes and activating the receptor with CCh or Oxo M. Several factors may account for this reduced efficacy. Firstly, activation of both receptors occurs upon stimulation with different compounds. Although the EC_50_ values for both are within a similar range *in vitro*, nematodes were incubated in lower concentrations of CNO compared to Oxo M or CCh due to its limited solubility. Moreover, it is well possible that different drug uptake levels or limited drug accessibility of CNO can explain the observed differences *in vivo*. Obviously, this potential limitation in drug accessibility needs to be kept in mind when using DREADDs for applications in different types of tissue in *C. elegans*. Secondly, lower expression levels of the DREADD compared to *gar-3* (Fig. 3) might also contribute to ceGAR-3Dq having a smaller effect on spicule protraction rate than GAR-3. However, despite these obvious limitations ceGAR-3Dq is useful for modulating G_q_-protein signalling in *C. elegans* for certain applications.

The use of this novel ceGAR-3Dq in particular and DREADDs in general in the model organism *C. elegans* is highly advantageous for various applications. It can be employed for studying the impact of G-protein pathways in distinct cell-types or organs on the whole organism, an approach for which mammalian systems sometimes are too complex. Many cell-specific promoters have been described and offer a platform to express DREADDs in specific tissues. Further, it is highly suitable to manipulate the activity of certain cell types with neuroscience being one potential field of application. The neuronal network of *C. elegans* offers an ideal system for studies on neuronal networks and connectomes as it is well characterised. A hermaphrodite contains 302 neurons, which form about 7,000 chemical synapses^40^. Probing specific populations of neurons and stimulating or inhibiting their activity will help understanding neuronal networks and subsequently, for instance animal behaviour. So far, optogenetic tools have been employed to address these topics^12^,^13^. DREADDs are a powerful alternative to this technique as they allow for a dose-dependent modulation of signalling activity.

In summary, our results show that DREADDs are a functional tool in the nematode *C. elegans*. The newly designed nematode-specific DREADD ceGAR-3Dq based on the G protein-coupled acetylcholine receptor GAR-3 activates a G_q_ protein-signalling cascade and is able to modulate related pathways *in vivo*. This receptor is exclusively stimulated by the inert compound CNO and thus, does not interfere with any physiological receptors. This pharmacogenetic toolbox now established in *C. elegans* offers a plethora of areas of application. To fully explore the entire experimental potential of DREADDs in *C. elegans* future analyses will need to focus on the design of nematode DREADDs specific for activating other G protein cascades such as G_s_ and G_i_ protein-signalling pathways.

## Methods

### Materials

Carbamylcholine chloride (carbachol, CCh), Oxotremorine M (Oxo M), forskolin, 3-isobutyl-1-methylxanthine (IBMX), aldicarb, and standard chemicals were purchased from Sigma Aldrich. Clozapine-N-Oxide (CNO) was obtained from the National Institutes of Health (Bethesda, MD) as part of the Rapid Access to Investigative Drug Program funded by the National Institute of Neurological Disorders and Stroke. Substances applied were dissolved in the media or buffer the according experiment was performed in.

### Generation of plasmids and transgenes

GAR-3b constructs for *in vitro* assays were generated by cloning *gar-3b* cDNA into the mammalian expression vector pcDNA5FRT and contained an N-terminal HA-tag which does not alter functional properties of GPCRs (pSP109)^41^. *cegar-3Dq* was engineered by introducing the two point mutations Y146C/A237G into pSP109 (pSP112). For generation of constructs for *in vivo* analyses recombineering was employed^42^,^43^. g*ar-3::yfp* (genomic) inserted behind the *gar-3* promoter (pYL9)^16^ was a kind gift from Dr. Rene Garcia, Texas A&M University. The construct containing the *C. elegans*-specific DREADD *cegar-3Dq* downstream the *gar-3* promoter (pSP110) was generated by inserting the two point mutations (Y146C/A237G) into the genomic sequence of *gar-3* in the vector pYL9 (kind gift from Dr. Rene Garcia, Texas A&M University)^16^. Constructs comprising *gar-3* promoter-driven mammalian DREADDs (pSP106, pSP108, pSP114) and rat M3R (pSP104) were cloned by exchanging *gar-3* for the cDNA of the respective receptor in pYL9. For details see Supplementary Methods.

### Cell culture

CHO-K1 and COS-7 cells were purchased from Leibniz Institute DSMZ – German Collection of Microorganisms and Cell Culture GmbH (CHO-K1: No. ACC 110, COS-7: No. ACC 60), HEK-GT cells were obtained from ThermoFisher Scientific (GripTite 293 MSR Cell Line, No. R79507). All cells were grown in F12 media or Dulbecco's modified Eagle medium (DMEM) supplemented with 10% fetal bovine serum, 100 U/ml penicillin, and 100 μg/ml streptomycin at 37 °C in a humidified incubator with 7% CO_2_. Lipofectamine 2000 (Life Technologies) was used for transient transfection according to the manufactures’ recommendations.

### Dynamic mass redistribution

Dynamic mass redistribution measurements are a label free assay system to study GPCR properties in living cells^44^. Thus, COS-7 cells were seeded into T25 flask 16 to 24 hours prior to transfection with 3 μg plasmid DNA and 7.5 μl Lipofectamine. The next day, 6,000 cells were split into one well of an Epic 384-Well Fibronectin-Coated Microplate (Corning, Kaiserslautern, Germany). DMR measurements were carried out at a Corning Epic label-free detection platform. CCh and CNO were dissolved in HBSS buffer at different concentrations. For equilibration, cells incubated with HBSS buffer for 2 hours, followed by a baseline recording for 5 minutes. After adding CCh and CNO DMR was recorded for up to 75 minutes. To determine G protein-coupling specificity cells were incubated either with pertussis (100 ng/ml) or cholera toxin (1 μg/ml) overnight prior to DMR recordings.

### IP accumulation assay

To measure inositol phosphate (IP) formation COS-7 cells were split into 12-well plates (1.5 × 10^5^ cells/well) and transfected with a total amount of 0.5 μg of plasmid DNA and 1.5 μl Lipofectamine per well. Subsequently, cells were incubated with 2 μCi/ml of myo-^3^H-inositol (18.6 Ci/mmol, Perkin Elmer) for 18 hours. Thereafter, cells were washed once with serum-free DMEM containing 10 mM LiCl followed by incubation with test compounds for 30 minutes at 37 °C. Intracellular IP levels were determined by anion-exchange chromatography as described previously^45^.

### Fluorometric Calcium measurements

COS-7 cells were split into T25-flasks (1.2 × 10^6^) and transfected with 3 μg plasmid DNA and 7.5 μl Lipofectamine the next day. 48 hours past transfection, cells were detached using Versene and labelled in DMEM with 4 μM Fluo-4 AM (Molecular probes) for 30 minutes at 37 °C. Free dye was removed by centrifugation, and cell suspensions were re-suspended into HBS buffer (132 mM NaCl, 10 mM HEPES, 6 mM KCl, 5.5 mM glucose, and 1 mM MgCl_2_, adjusted to pH 7.4 with NaOH). The cell suspension was dispensed into black, clear-bottom 384 microwell plates (Corning, Kaiserslautern, Germany) with 45.000 cells per well. Fluorescence measurements were performed in a two-step protocol using a fluorescence imaging plate reader and a robotic liquid handling station (Freedom Evo 150, Tecan, Männedorf, Switzerland). Fluorescence intensity was corrected for the background and normalized to the initial intensities (F/F_0_).

### *C. elegans* strains

*C. elegans* strains were cultured and manipulated according to standard protocols^46^. Wild-type worms were *C. elegans* variety Bristol, N2 and grown at 22 °C. The allele *gar-3*(*gk305*) was generated by the *C. elegans* gene knockout consortium. The strain *pha-1*(*e2123*)*; him-5* (*e1490*) *gar-3*(*gk305*) was previously described (kind gift from Dr. Rene Garcia, Texas A&M University)^16^ and kept at 15 °C. The transgenes *aprEx183*[*gar-3::yfp* (*pYL9*) *pha-1*(+) *pBSK*]*, aprEx184*[*cegar-3Dq::yfp* (*pSP110*) *pha-1*(+) *pBSK*]*, aprEx186*[*rM3R::yfp* (*pSP104*) *pha-1*(+) *pBSK*], *aprEx187*[*rM3Dq::yfp* (*pSP106*) *pha-1*(+)*pBSK*] *aprEx188*[*rM3Ds::yfp* (*pSP108*) *pha-1*(+) *pBSK*] and *aprEx189*[*hM4Di::yfp* (*pSP114*) *pha-1*(+) *pBSK*] were generated for this study (for details see Supplementary Methods) and cultivated at 25 °C.

### Spicule protraction assay

24 hours prior to conducting the assay, wild-type L4 males were put separately onto NGM agar plates containing *Escherichia coli* OP50. Subsequently, males were mounted onto a 2% agarose pad and a 100 μl of 2 mM CNO, 100 mM Oxo M, 10 mM CCh or H_2_O, respectively, were applied. Males were scored for spicule protraction using a Leica M165FC microscope.

Nematodes with spicules partially or fully protracted were scored positive. Protraction rates were calculated in relation to the total number of males investigated.

### Microscopy

For analysis of transgene expression adult males were mounted in M9 containing 250 μM levamisole onto a 2% agarose pad. Differential interference contrast (DIC) and confocal fluorescent images were collected with an Olympus Fluoview FV1000 setup. Fluorescence signals were quantified by intensity analysis using ImageJ software^47^.

### Statistical analyses

Statistical significance of assay data from spicule protraction assays was determined using a Fisher’s exact test for each genotype and condition. For statistical analyses of brood size, individuals reaching adulthood, lifespan, locomotion, pharyngeal pumping, egg laying, sensitivity to aldicarb, and fluorescent intensity calculations a student’s t-test was performed.

## Additional Information

**How to cite this article**: Prömel, S. *et al.* Deciphering and modulating G protein signalling in *C. elegans* using the DREADD technology. *Sci. Rep.* **6**, 28901; doi: 10.1038/srep28901 (2016).

## Supplementary Material

Supplementary Information
