# Supplementary material for: Deciphering and modulating G protein signalling in C. elegans using the DREADD technology
Source: Sci Rep. 2016 Jul 27;6:28901. doi: 10.1038/srep28901 (PMC4962097; doi:10.1038/srep28901)
Supplement: Supplementary Information [file srep28901-s1.pdf]

Supplementary Information

Deciphering and modulating G protein signalling in *C. elegans* using the DREADD technology

Simone Prömel, Franziska Fiedler, Claudia Binder, Jana Winkler, Torsten Schöneberg, and Doreen Thor

Supplementary Items

Supplementary Figures and Tables

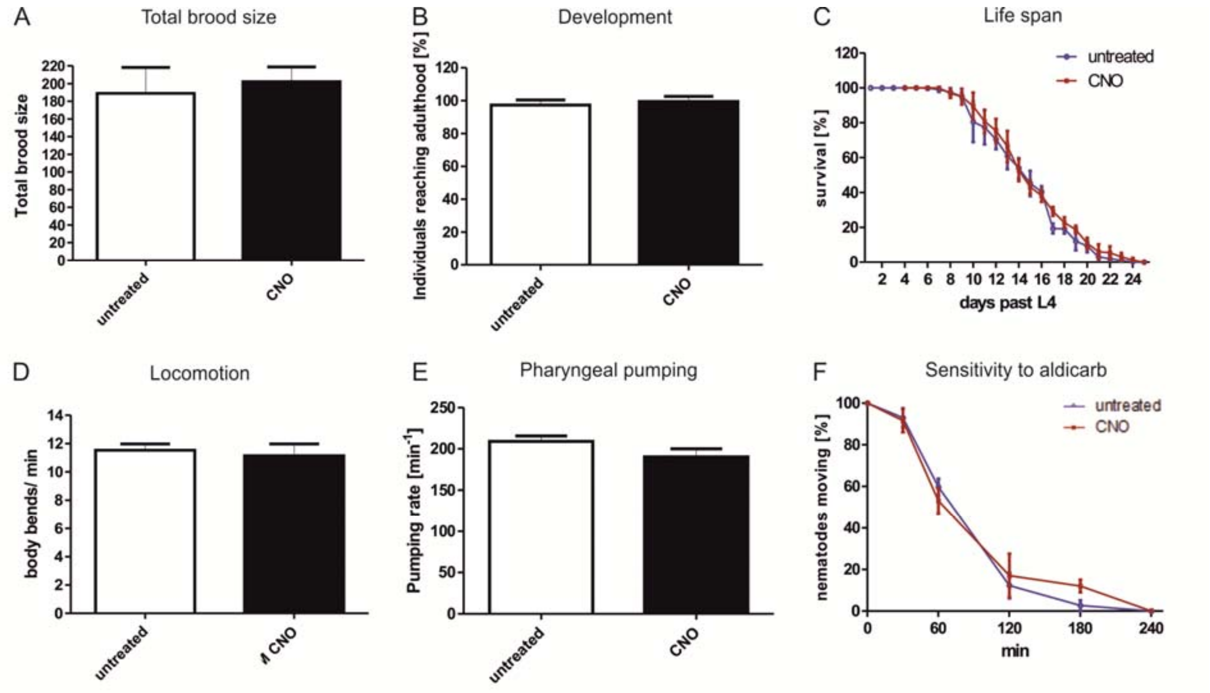

G Egg laying

condition	bag of worms	eggs inside the uterus	eggs laid /5 hours	sets
untreated	2/90	11.8 ± 1.4 (n = 61)	6.6 ± 1.2 (n = 61)	3
1 mM CNO	1/79	11.7 ± 2.0 (n = 58)	8.3 ± 1.1 (n = 58)	3
2 mM CNO	2/76	11.4 ± 1.6 (n = 52)	6.1 ± 0.7 (n = 52)	3

Supplementary Figure 1: The inert drug CNO does not have any detrimental effects on *C. elegans*. (A) Total brood size of wild-type hermaphrodites treated with 2 mM CNO or M9 (untreated control). Single hermaphrodites were incubated in liquid culture containing *E. coli OP50* and transferred daily. Brood size was scored until egg laying ceased. n ≥ 20. (B) CNO does not have an effect on development to adult stages.

Hermaphrodites were cultivated from stage L4 onwards in liquid culture containing 2 mM CNO or M9 (untreated control) and *E. coli OP50*. Subsequently, layed eggs were placed into fresh liquid culture with and without CNO and individuals reaching adulthood were scored. Data are given as percentage of original egg number, $n \geq 200$. (C) Lifespan of wild-type nematodes is not influenced by CNO. L4 larvae were placed individually in liquid culture containing either M9 (untreated control) or 2 mM CNO with *E. coli OP50* and scored daily. Survival rates were determined as percentage of original number nematodes, $n \geq 15$. (D) Body bends per minute of wild-type nematodes stimulatd with 2 mM CNO are not different from untreated controls (M9). Single hermaphrodites were incubated in liquid culture containing *E. coli OP50*. After 24 hours nematodes were transferred to plates containing *E. coli OP50* and body bends per minute were scored. $n \geq 40$. (E) Pharyngeal pumping rates were determined in wild-type hermaphrodites treated with 2 mM CNO or M9 (untreated control) after 24 hours. Single hermaphrodites were incubated from stage L4 onwards in liquid culture containing *E. coli OP50* and pumps per minute counted on plates. $n \geq 55$. (F) Sensitivity to aldicarb was determined by measuring movement of young adult hermaphrodites over time in 4 mM aldicarb with 2 mM CNO or M9 (untreated control). Data are given as percentage of total nematode numer $n \geq 35$. (G) CNO has no effect on egg laying behaviour. Animals at L4 stage were incubated in liquid culture containing 1 mM CNO, 2 mM CNO or M9 (untreated control), respectively, and *E. coli OP50*. After 48 hours bag of worms formed within the nematodes were counted. For analysing eggs inside the uterus and eggs laid/5 hours egg laying was monitored for 5 hours after 24 hours of incubation in solutions containing the respective compound and *E. coli OP50*. Finally, the number of eggs inside each nematode was determined after lysing the nematodes.
All data are shown as mean ± SD.

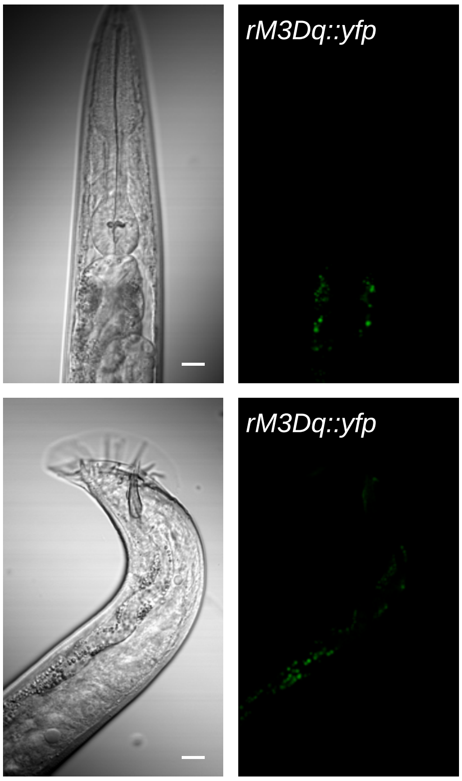

Supplementary Figure 2: Rat DREADD expression cannot be detected in *C. elegans*. Transgenic nematodes carrying *rM3Dq::yfp* driven by the *gar-3* promoter do not show any expression of the DREADD in the pharynx (top) and the male tail (bottom). Left: DIC, right: fluorescent image. Scale bar = 10 µm.

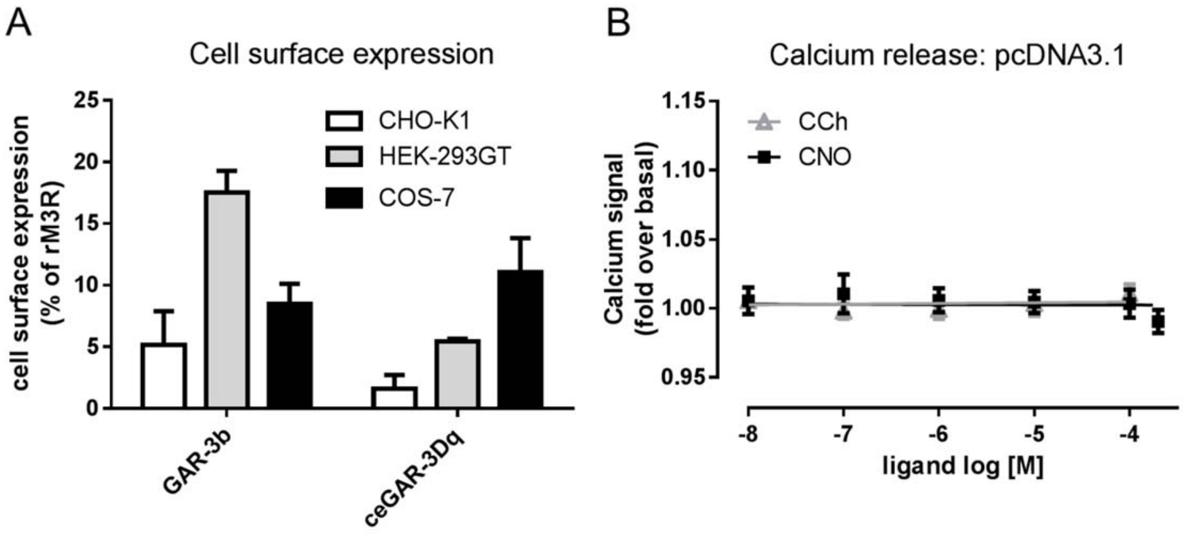

Supplementary Figure 3: Analysis of GAR-3b and ceGAR-3Dq properties. (A) Cell surface of GAR-3b and ceGAR-3Dq expression in commenly used cell lines were determind using an ELISA. Thereto, CHO-K1, HEK-293GT, and COS-7 cells were transfected with N-terminally tagged rM3R, GAR-3b, and ceGAR-3Dq. Thus, cell surface expression of rM3R was utilized as reference and set to 100 %. Background in colorimetric development was determined using mock-transfected cells. Given is the mean ± SD of 3 (CHO-K1 and HEK-293GT) to 7 (COS-7) independent experiments performed in triplicates. (B) Calcium release in mock-transfected COS-7 cells. Calcium release was measured using Fluo-4 labeled cells. Increasing concentrations of CCh and CNO do not trigger Calcium release in mock-transfected cells. Given is the mean ± SEM of 3 independent experiments performed in duplicates.

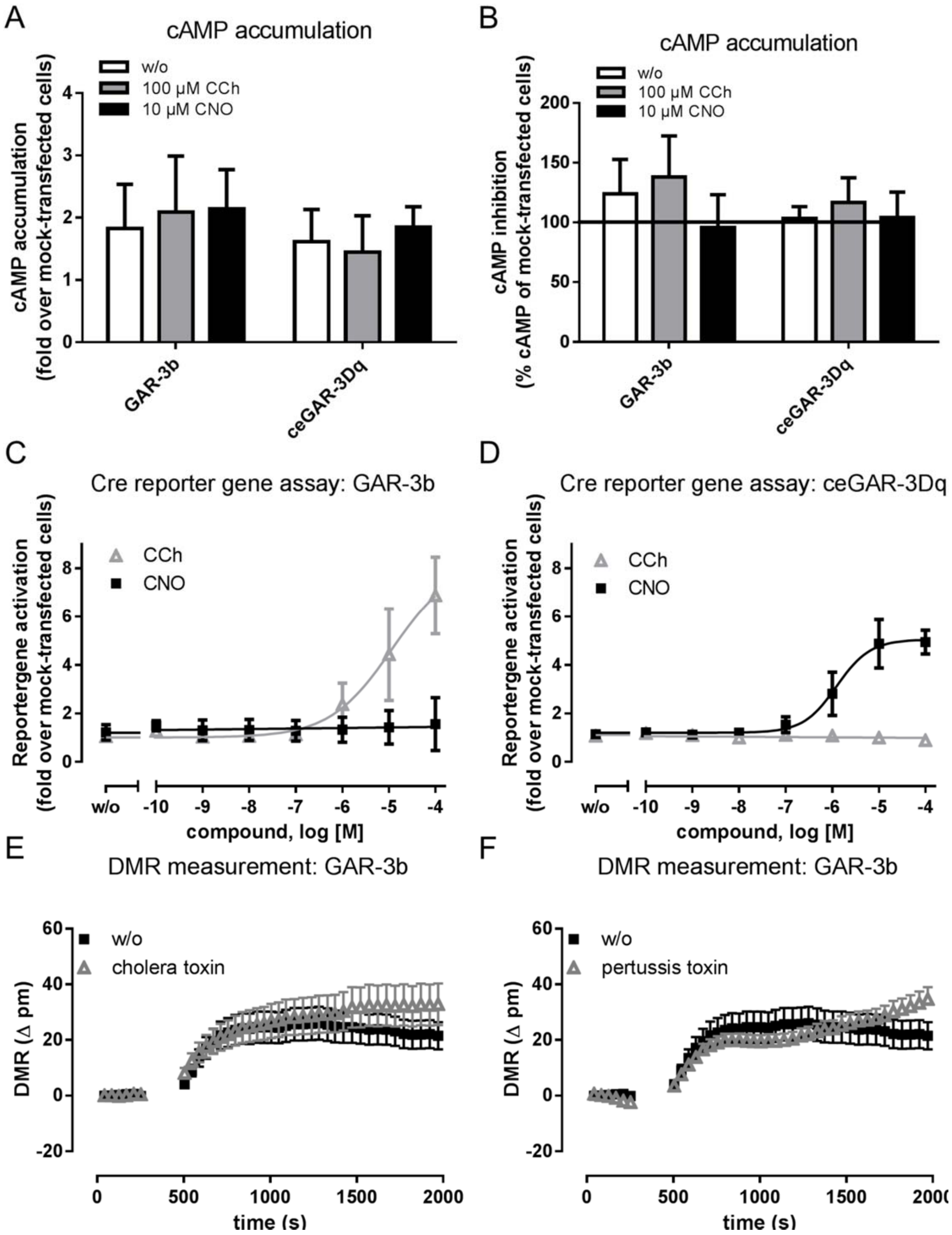

Supplementary Figure 4: Second messenger assays to study G_s- and G_i-protein coupling. (A) Transfected cells were incubated with media, 100 µM CCh, or 10 µM CNO for 30 minutes. Accumulation of cAMP was determined using the AlphaScreen Technology. (B) Transfected cells were pre-treated with 10 µM forskolin for 10 min and then incubated with media, 100 µM CCh, or 10 µM CNO. Given is the mean ± SD of three independent experiments performed in triplicates. (C/D) CRE-mediated reporter gene assays were performed with GAR-3b transfected (C) and ceGAR-3Dq transfected (D) cells. After incubation with CCh and CNO cells were lysed and reporter gene expression determined as a degree of cAMP formation. Given are the mean ± SD of three independent experiments performed in triplicates as fold over of unstimulated mock-transfected cells (93,111 ± 12,263 cps). (E/F) DMR measurements with CCh stimulated GAR-3b were performed with and

without cholera toxin (E) and pertussis toxin (F) to specifically inhibit G_s- and G_i-protein signalling, respectively. Given is the mean ± SD of two independent experiments performed in triplicates.

Supplementary Table 1: Protraction rate counts in male nematodes containing mammalian DREADD constructs (A) or the *C. elegans*-specific DREADD (B). Given is the mean ± SD, n ≥ 250. The data in (A) are the basis of the graph in Fig. 1E, the data in (B) are shown in the graph in Fig. 4F.

A

	H_2O	OxoM	CCh	CNO
**wild-type**	15.3 ± 3.9	82.5 ± 6.1	84.7 ± 7.1	23.5 ± 5.9
gar-3	15.0 ± 6.9	13.0 ± 5.8	13.9 ± 3.6	12.8 ± 2.4
gar-3; Ex[gar-3(+)]	12.3 ± 3.9	80.0 ± 5.6	78.4 ± 4.0	14.7 ± 6.2
gar-3; Ex[rM3Dq]	15.7 ± 3.8	14.1 ± 4.8	14.5 ± 8.5	16.4 ± 5.7
gar-3; Ex[rM3R]	15.2 ± 10.1	14.4 ± 4.2	11.8 ± 4.5	11.4 ± 6.0

B

	H_2O	OxoM	CCh	CNO
**wild-type**	16.9 ± 2.1	83.2 ± 3.3	76.3 ± 7.8	19.2 ± 8.9
gar-3	16.4 ± 5.7	10.6 ± 4.8	12.8 ± 2.4	12.8 ± 2.4
gar-3; Ex[gar-3(+)]	15.4 ± 3.7	71.3 ± 2.4	79.0 ± 6.3	11.9 ± 2.2
gar-3; Ex[cegar-3Dq]	15.9 ± 6.1	17.9 ± 11.6	14.6 ± 7.8	37.4 ± 6.3

Supplementary Table 2: Transformants and lines obtain after injection of mammalian DREADDs.

	rM3R	rM3Dq	rM3Ds	hM4Di
**injections**	29	31	65	37
**transformants**	82	132	124	97
**lines carrying transgene**	7	6	7	5

Supplementary Table 3: pEC50 values for rM3R, GAR-3b, and ceGAR-3Dq determined in *in vitro* assays. Given is the mean ± SD of three independent experiments performed in duplicates or triplicates.

	rM3R / CCh	GAR-3b / CCh	ceGAR-3Dq / CNO
**DMR measurement**	-7.49 ± 0.13	-5.24 ± 0.56	-5.46 ± 0.43
**Calcium release**	-7.06 ± 0.21	-4.79 ± 0.26	-6.21 ± 0.99
**CRE reporter gene assay**	-5.94 ± 0.26	-5.31 ± 0.53	-5.80 ± 0.65

Supplementary Table 4: Sequence of primers used to generate constructs presented in the study.

Primer	Sequence 5'-3'
RASSL_10R	CGTGTACAGAATGCACATAATTGTAACAGGAAGATAAAATCCAGCAACC
RASSL_11F	TAAATTTTCAGACATACACCATTATTACTCCTCCTGCACATTTATTAATCATGACCTTGCACAGTAACAGTACAACCTCGCCT
RASSL_12R	TTAGTATCATTCGAAACATACCTTTGGGTCCTTTGGCCAATCCCGGGGATCCTCTAGGCAAGGCCTGCTCCGGCACTCGCTTGTGAAA
RASSL_13F	TTGGCCAAAGGACCCAAAGGTATGTTTCGAATGATACTAAGAAGTTCCTATACTTTCTAGAGAATAGGAA
RASSL_14R	TTTTTCTACCGGTACCCTCAAGGGTCCTCCTGAAAATGTTCTATGTTATGTGGAGGCTACCATGGAGAAGTTACTATTCC
RASSL_17R	TTAGTATCATTCGAAACATACCTTTGGGTCCTTTGGCCAATCCCGGGGATCCTCTAGGGGTGGCGACCGGTAAGGCCTGCTCCGGCAC
RASSL_18F	TAAATTTTCAGACATACACCATTATTACTCCTCCTGCACATTTATTAATCATGATGGCCAACTTCACACCTGTCAATGGCAGC
RASSL_19R	TTAGTATCATTCGAAACATACCTTTGGGTCCTTTGGCCAATCCCGGGGATCCTCTAGGCTACCTGGCAGTGCCGATGTTCCGATACTG
RASSL_34F	AAATACTTTCTCGATGCACTGTTCACATTAGTGAAATAAAATCCACCGGATCTAGATAACTGATCAGCTT
RASSL_35R	ATGTGAACATGGGTATTGATATGACACCTGAAATATGATACCTCTGAGCTATTCCAGAAGTAGTGAGGAG
RASSL_36F	GCGGCCGCTCGAGTCTAGAGGGCC
RASSL_37R	GGCGTAGTCGGGGACGTCGTAGGG
RASSL_38F	CAGTCCTCTTCGTTGGGGAATGC
RASSL_39R	CTAGTTGCGTCGGACATATCCCTG
RASSL_42F	GCAGATATTGCAATTGGTGAGTGG
RASSL_43R	ATCGATGCATAACCAGAACTGGC
RASSL_44F	TTTTATCTTCCTGTTACAATTATGTGC
RASSL_45R	AAAATCATAACAATTTGAAAATCG
RASSL_46F	GTGTCATATCAATACCCATGTTCAC
RASSL_49R	GTCATATCAATACCCATGTTCACA
RASSL_50R	ACCAATTGCAATATCTGCGACGGC
RASSL_51F	TCTCGTGTACAGAATGCACATAAT

Supplementary Methods

Cell surface expression
To assess receptor expression 3 x 10^4 COS-7 cells, 4 x 10^4 CHO-K1 cells, and 6 x 10^4 HEK-293GT cells, respectively, were split into 48-well plates and transfected with 0.2 µg DNA and 0.5 µl lipofectamine per well. In brief, after fixating the cells with 4% formaldehyde and blocking with 10% fetal bovine serum (FBS) in DMEM, cells were incubated with peroxidase-coupled-anti-HA-antibody (Roche, 1 µg/ml in DMEM with 10% FBS) at room temperature for 1 hour, followed by extensive washing. Enzymatic reaction was carried out at room temperature in the presence of H_2O_2 and o-phenylenediamine. The reaction was stopped by adding 50 µl of 50 mM Na_2SO_3 in 1 M HCl. Color development was measured at 492 nm and 620 nm using a Sunrise™ plate reader (Tecan).

cAMP accumulation assay
To assess G_s- or G_i-protein coupling, cAMP accumulation assays were performed in 48-well plates. One day after transfection cells were stimulated with 100 µM CCh or 10 µM CNO in DMEM containing 1 mM IBMX for 30 min at 37 °C. For measuring G_i-protein activation cells were pre-stimulated with 10 µM forskolin for 10 minutes followed by incubation with CCh or CNO in forskolin-containing media. Reactions were stopped by aspiration of media and cells were lysed in 50 µl of lysis buffer containing 5 mM HEPES, 0.3 % Tween, 0.1 % BSA, and 1 mM IBMX. cAMP content of cell extracts was determined by a non-radioactive cAMP accumulation assay based on the ALPHAScreen™ technology according to manufacturer's protocol (AlphaScreen® cAMP Assay Kit, Perkin Elmer).

CRE-SeAP reporter gene assay
For the CRE-SeAP (secreted alkaline phosphatase) reporter gene assay COS-7 cells were seeded into T25 flasks and co-transfected with receptor expression plasmid and CRE-SeAP reporter plasmid (Clontech). One day after transfection cells were split into 96-well plates and serum-free media with increasing concentrations of CCh or CNO was added the following day. Cells were incubated for 24 hours at 37°C followed by cell lysis at 65–70°C for 2 hours. An aliquot of the supernatant from each well was then incubated (2–5 minutes, 21°C) with an equal volume of 1.2 mM 4-methylumbelliferyl phosphate in 2 M diethanolamine bicarbonate with 1 mM $MgCl_2$ and 4.5 mg/ml L-homoarginine (pH 10). Fluorescence was measured with a Victor 2–1420 Multilabel counter (Perkin Elmer).

Generation of plasmids and transgenes
Plasmids for *in vitro* functional assays were generated by cloning techniques, constructs for *in vivo* analyses were engineered using recombineering [1]. For primer sequences see Supplementary Tab. 4.

gar-3b in pcDNA5FRT (pSP109)
The sequence of *gar-3b* was amplified from a *C. elegans* cDNA library with phosphorylated primers RASSL_38F/RASSL_39R. The vector pcDNA5FRT containing an N-terminal hemagglutinin (HA) epitope tag was amplified using primers RASSL_36F/RASSL_37R, digested with *Dpn*I and ligated with *gar-3*.

cegar-3Dq in pcDNA5FRT (pSP112)
Construct pSP109 was used as a basis for *cegar-3Dq* in pcDNA5FRT. Using an outward PCR with phosphorylated primers RASSL_50R/RASSL_52F the plasmid was amplified except the region in which the point mutations were to be inserted. The region containing the point mutations was amplified from pSP110 with primers RASSL_49R/RASSL_51F and subsequently ligated with the amplified vector.

cegar-3Dq::yfp (pSP110)
To generate the point mutations for pSP110, a FRT-kanR-FRT cassette amplified from pIGCN21 [2] with primers RASSL_34F/RASSL_35R which contain overhangs with homologies to pYL9. The cassette was recombineered into intron 3 of pYL9 using electrocompetent SW105 cells with heat-induced recombinase of the λ-Red recombinase system [3]. Subsequently, three fragments were generated: (1) a FRT-kanR-FRT cassette with flanking regions from the newly generated cassette in pYL9 (primers RASSL_42F/RASSL_43R), (2) a fragment introducing the two point mutations (TAT to TGC change for Y146C and a GCA to GGA change for A237G) with primers RASSL_46F/RASSL_10R and (3) a fragment of the region behind the point mutations in pYL9 (primers RASSL_44F/RASSL_45R). All three fragments were fused with fragments (1) and (2) being subject to an overlap-extension PCR first (primers RASSL_46F/RASSL_45R) and subsequently fusion of the resulting product to (3) using primers RASSL_42F/ RASSL_45R. The obtained fragment was inserted into pYL9 by

recombineering using the recombinase of the λ-Red recombinase system [3] in electrocompetent SW105 cells. The selection cassette was subsequently removed using activated FRT recombinases in SW105 cells.

pgar-3::M3R::yfp (pSP104), *pgar-3:: rM3Dq::yfp* (pSP106), *pgar-3:: rM3Ds::yfp* (pSP108), *pgar-3:: hM4Di::yfp* (pSP114)

To generate constructs containing mammalian DREADD or rat M3R, respectively, a recombineering targeting cassette consisting of two parts was generated. A fragment containing the cDNA of the respective receptor was amplified using primers RASSL_11F/RASSL_12R (rM3R), RASSL_11F/RASSL_12R (rM3Dq), RASSL_11F/RASSL_17R (rM3Ds) or RASSL_18F/RASSL_19R (hM4Di) with the respective forward primer containing overhangs homologous to the *gar-3* promoter region in pYL9. In parallel, a FRT-kanR-FRT cassette was amplified from pIGCN21 [2] (primers RASSL_13F/RASSL_14R) with the reverse primer containing a homology to the sequence of *yfp* in pYL9. To ensure ligation of this cassette to the 3´end of the respective fragment, the forward primer of the cassette was phosphorylated beforehand. Ligation was conducted using an 1: 1 molar ratio of both fragments. The ligation product was subject to another PCR amplifying the complete ligated region applying primers RASSL_11F/ RASSL_14R (rM3R), RASSL_11F/RASSL_14R (rM3Dq), RASSL_11F/RASSL_14R (rM3Ds) or RASSL_18F/RASSL_14R (hM4Di). The resulting cassette was recombineered into pYL9 using electrocompetent SW105 cells with heat-induced recombinase of the λ-Red recombinase system [3]. The selection cassette was subsequently removed using SW105 cells containing activated FRT recombinases.

Generation of transgenic lines

All transgenic strains with stably transmitting extrachromosomal arrays were generated by DNA microinjection as described [4]. Plasmids were injected at a concentration of 30 ng/µl together with the coinjection marker pBX1 (*pha-1 (+)*) (100 ng/µl) and pBluescript II SK+ vector DNA (Stratagene) as stuffer DNA to achieve a final concentration of 150 ng/µl. DNA was injected into the syncytical gonad of *pha-1(e2123); him-5 (e1490) gar-3(gk305)* hermaphrodites. Transgenic progeny were isolated, stable lines selected and checked for the presence of the transgene by PCR. Multiple independent transgenic lines were established for each transgene tested.

Brood size, development and lifespan assays

L4 hermaphrodites were placed separately into the wells of a flat-bottom 72-well Terasaki plate (Greiner Bio One) containing 2 mM CNO in M9 or M9 (control), respectively, with heat-killed *E. coli OP50* (75°C, 30 minutes), and allowed to lay eggs at 22°C. Every 24 hours the mother animal was transferred to a fresh well and the brood size determined. Mothers were subsequently transferred daily, monitored until death and the number of dead and alive worms scored each day. Offspring were incubated at 22°C and the number of adult animals was scored 48 hours after the mother was removed. All experiments were conducted at least in triplicate.

Locomotion assay

For elucidating locomotion, body bends per minute determined. 24 hours prior to the assay wild-type L4 hermaphrodites were placed separately into the wells of a flat-bottom 72-well Terasaki plate (Greiner Bio One) containing 2 mM CNO or M9 (control), respectively, and heat-killed *E. coli OP50* (75°C, 30 minutes) at 22°C. Body bends were scored on NGM plates seeded with *E. coli OP50*. A body bend was defined as the part of the worm just behind the pharynx reaching a maximum bend in the opposite direction from the bend prevous bend. The number of body bends were minute was determined. Experiments were conducted in three independent assays.

Pharyngeal pumping assay

For determining pharyngeal pumping rates, 24 hours prior to the assay wild-type L4 hermaphrodites were placed separately into the wells of a flat-bottom 72-well Terasaki plate (Greiner Bio One) containing 2 mM CNO or M9 (control), respectively, and heat-killed *E. coli OP50* (75°C, 30 minutes). Nematodes were incubated at 22°C. Pharyngeal pumps were counted visually on NGM plates seeded with *E. coli OP50* using a Leica M165FC microscope. Analyses were conducted in three independent assays.

Aldicarb assay

Wild-type L4 hermaphrodites were transferred into the wells of a flat-bottom 72-well Terasaki plate (Greiner Bio One) containing 2 mM CNO in M9 or M9 (control), respectively, with heat-killed *E. coli OP50* (75°C, 30 minutes), and incubated at 22°C for 24 hours. Subsequently, they were placed separately into wells of a flat-bottom 72-well Terasaki plate. Worms previously incubated in 2 mM CNO were transferred into well containing 2 mM CNO and 4 mM aldicarb in M9 (aldicarb stock 100 mM in ethanol), nematodes previously kept in M9 were placed into wells with 4 mM aldicarb in M9. Movement of worms was monitored over time and nematodes moving vs. not moving were scored visually after 30, 60, 120, and 240 minutes. Analyses were conducted in four independent experiments.

Egg laying assays

Wild-type L4 larvae were allowed to mature overnight in wells of a flat-bottom 72-well Terasaki plate (Greiner Bio One) containing M9, 2mM CNO, respectively, and heat killed *E. coli OP50* (75°C, 30 minutes). Bags of worms within a hermaphrodite were scored after 48 hours. For elucidation of egg laying over time, nematodes were transferred to individual wells containing the respective drug and heat-killed *E. coli OP50*. After 5 hours laid eggs were counted. Subsequently, the same nematodes were transferred in wells of a flat-bottom 72-well Terasaki plate with a 15% sodium hypochlorite solution slightly shaking to dissolve the worms but keep the eggs. After 10 minutes eggs inside the uterus were counted using a Leica M165FC microscope. All experiments were conducted at least in triplicate.

Supplementary References

[1] Dolphin, C. T. and Hope, I. A., Caenorhabditis elegans reporter fusion genes generated by seamless modification of large genomic DNA clones. *Nucleic Acids Res* **34** (9), e72 (2006); Tursun, B., Cochella, L., Carrera, I., and Hobert, O., A toolkit and robust pipeline for the generation of fosmid-based reporter genes in C. elegans. *PLoS One* **4** (3), e4625 (2009).

[2] Lee, E. C. et al., A highly efficient Escherichia coli-based chromosome engineering system adapted for recombinogenic targeting and subcloning of BAC DNA. *Genomics* **73** (1), 56 (2001).

[3] Warming, S. et al., Simple and highly efficient BAC recombineering using galK selection. *Nucleic Acids Res* **33** (4), e36 (2005).

[4] Mello, C. and Fire, A., DNA transformation. *Methods Cell Biol* **48**, 451 (1995); Mello, C. C., Kramer, J. M., Stinchcomb, D., and Ambros, V., Efficient gene transfer in C.elegans: extrachromosomal maintenance and integration of transforming sequences. *Embo J* **10** (12), 3959 (1991).
